# Self-reference and random sampling approach for label-free identification of DNA composition using plasmonic nanomaterials

**DOI:** 10.1038/s41598-018-25444-2

**Published:** 2018-05-09

**Authors:** Lindsay M. Freeman, Lin Pang, Yeshaiahu Fainman

**Affiliations:** Department of Electrical and Computer Engineering, University of California San Diego, 9500 Gilman Drive, La Jolla, California 92093-0407 USA

## Abstract

The analysis of DNA has led to revolutionary advancements in the fields of medical diagnostics, genomics, prenatal screening, and forensic science, with the global DNA testing market expected to reach revenues of USD 10.04 billion per year by 2020. However, the current methods for DNA analysis remain dependent on the necessity for fluorophores or conjugated proteins, leading to high costs associated with consumable materials and manual labor. Here, we demonstrate a potential label-free DNA composition detection method using surface-enhanced Raman spectroscopy (SERS) in which we identify the composition of cytosine and adenine within single strands of DNA. This approach depends on the fact that there is one phosphate backbone per nucleotide, which we use as a reference to compensate for systematic measurement variations. We utilize plasmonic nanomaterials with random Raman sampling to perform label-free detection of the nucleotide composition within DNA strands, generating a calibration curve from standard samples of DNA and demonstrating the capability of resolving the nucleotide composition. The work represents an innovative way for detection of the DNA composition within DNA strands without the necessity of attached labels, offering a highly sensitive and reproducible method that factors in random sampling to minimize error.

## Introduction

The evaluation of DNA without costly DNA sequencing^1,2^ or labeling methods^3^ is imperative to reducing healthcare costs, so efforts must be made to develop DNA screening technologies that rely on cost reduction technology and scientific simplicity. Here, we examine a new label-free DNA detection method using surface-enhanced Raman spectroscopy (SERS)^4–6^, an optical technique that probes molecules directly rather than relying on the use of labels. SERS has been proposed as a label-free detection method of DNA^7–9^ due to the rich molecular information that Raman scattering reveals^10–14^, but previous attempts have suffered from the inability to quantify information related to the composition of DNA^12,15^. We report an approach to accurately identify the composition of the DNA bases adenine and cytosine benefiting from the fact that each base shares the same phosphate backbone structure, which can be treated as a reference to extract the base composition of adenine and cytosine within the DNA strands. This self-reference approach would be immune to varying experimental conditions due to the normalization of the data, as compared to other experiments that rely on absolute intensity measurements in which the signal varies as conditions change.

The self-reference approach provides a calculated ratio of the nucleotide signal with respect to the phosphate backbone, regardless of the variation in experimental conditions. Additionally, a highly accurate and precise statistical distribution can be maintained when a large number of experiments are conducted with uncontrollable environmental conditions via random sampling, which combined with the self-reference approach can be employed to determine the nucleotide composition in the DNA strand. As an example, we employ SERS as a demonstration of the self-reference approach, in which the nucleotide composition is acquired from several samples of synthesized DNA strands composed of varying mixtures of adenine and cytosine. In this case, a high concentration of adenine and cytosine is used in order to maximize the SERS signal at short acquisition times. Additionally, as this is the first demonstration of this technique, we selected adenine and cytosine due to the fact that their prominent Raman modes do not overlap with each other or the phosphate backbone mode, and thus are the ideal bases to utilize as a proof of concept. Future studies will take into account the additional bases of guanine and thymine; however, as a preliminary demonstration, only adenine and cytosine were analyzed in this work.

To demonstrate the simplicity of a self-reference approach on readily available substrates, we employ low cost random silver films as plasmonic resonators that localize the electromagnetic field for improved optical signal generation^16–19^. The self-reference of the phosphate backbone provides statistical bias to eliminate the nature of the experimental variation, which in our case, is caused by the DNA orientation on the metal surface, random distribution of plasmonic particles, and variable signal to noise ratio. The random silver films used in this work are fabricated in an uncontrolled manner, and thus have an uneven distribution of plasmonic hot-spots. However, as demonstrated in this work, the variation in the electromagnetic field enhancement is significantly reduced by taking into account hundreds of Raman spectra that minimize the input noise into our system.

**Figure 1:**
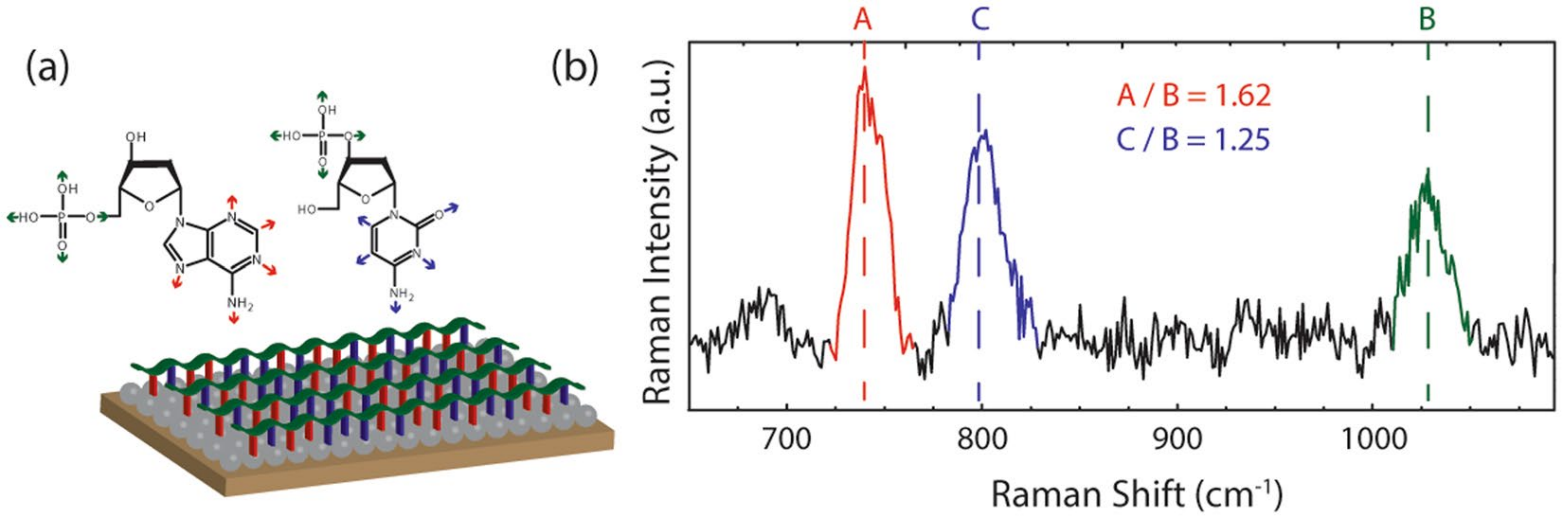
Normalization procedure for DNA composition detection using Raman spectroscopy. (**a**) Illustration of ssDNA functionalized to random silver films on mica showing the ring-breathing-modes of adenine (red), cytosine (blue) and the phosphate backbone mode of DNA (green). (**b**) Proposed normalization procedure in which the intensity of the adenine ring-breathing-mode (508 a.u.), of the cytosine ring-breathing-mode (394 a.u.), and of the backbone mode (314 a.u.) are used to calculate the A/B ratio (1.62) and C/B ratio (1.25).

Because measured SERS spectra vary across similar samples of interest and thus offer poor quantitative information, based on the self-reference strategy discussed above, we introduce a normalization procedure in which the ratio of the ring-breathing-modes of the nucleotides to the backbone mode of the DNA is found for each spectrum. When measuring various compositions of nucleotides within DNA strands using SERS, the number of each nucleotide will vary while the amount of DNA backbone will remain constant (Fig. 1a). Thus, the normalization procedure is dependent on the backbone mode as it stays constant throughout the spectral measurements. As shown in Fig. 1b, the strong Raman spectral features of the ssDNA are the ring-breathing-modes for adenine (735 cm^−1^) and cytosine (795 cm^−1^) and the phosphate backbone mode of the DNA strand (1030 cm^−1^). The intensity counts for each mode are used to calculate the A/B and C/B ratios and is the standard metric used to compare different compositions of DNA strands. The current work demonstrates the feasibility of a self-reference approach for the identification of nucleotide composition within DNA using a simplified model, in which synthesized single strands of DNA composed of adenine and cytosine are functionalized to random silver films and the SERS spectra are acquired.

## Results

**Figure 2:**
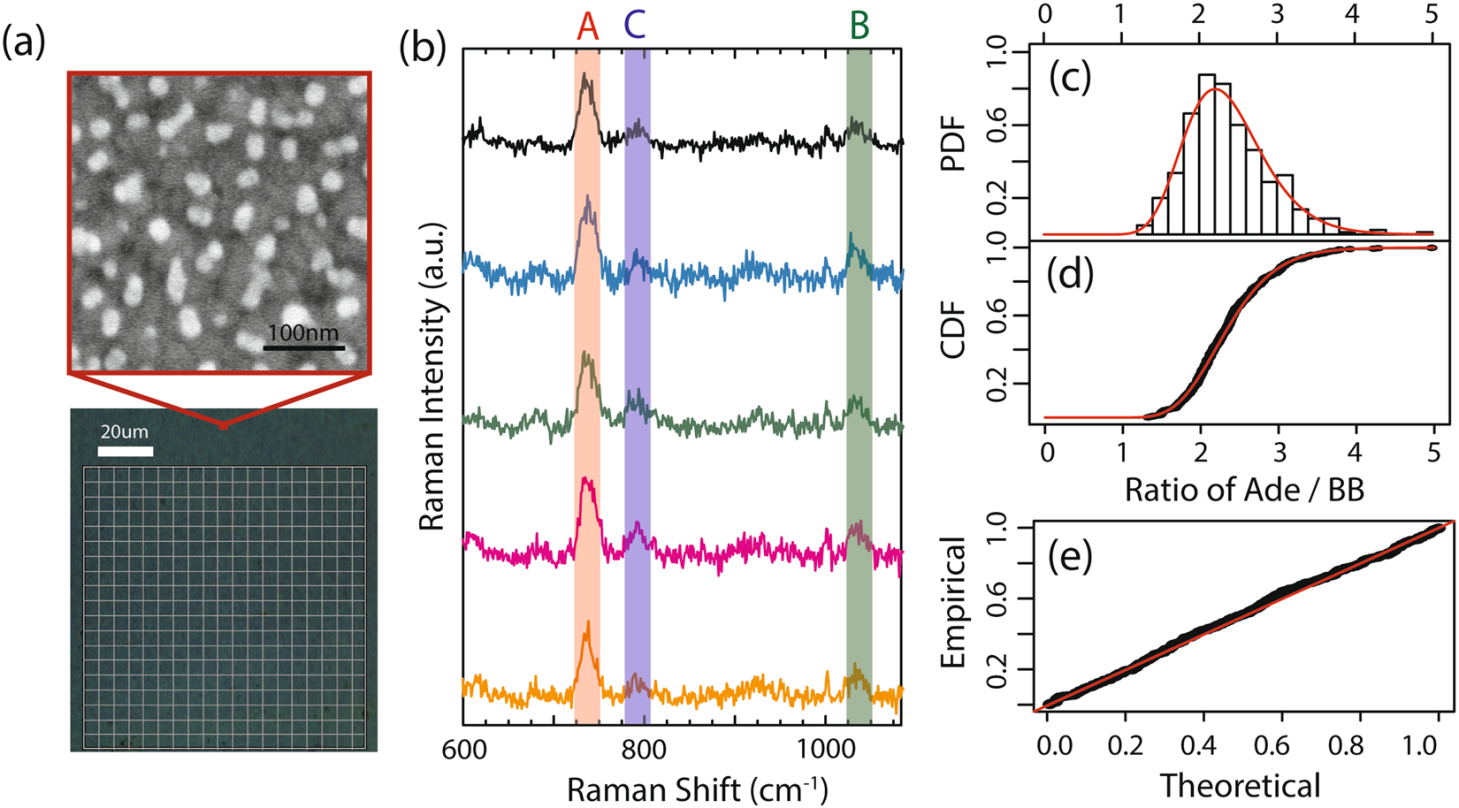
Random Raman mapping of ssDNA funtionalized to silver films. (**a**) Scanning electron microscope image of random silver films grown on mica and bright field image of ssDNA functionalized to random silver films on mica with the outline of the Raman map, in which 400 measurements are acquired in an approximately 100 micron by 100 micron area. (**b**) Example Raman spectra for 5 measurements of 75% A/25% C ssDNA mixture functionalized to random silver films, with the adenine ring-breathing-mode (red, 735 cm^−1^), cytosine ring-breathing-mode (blue, 795 cm^−1^), and backbone mode (green, 1035 cm^−1^) labeled. (**c**) Probability density function (PDF) of the A/B ratio for the 75% A/25% C mixture, showing the best fit lognormal distribution with a lognormal median at A/B = 2.36. (**d**) Cumulative distribution function (CDF) of the 400 Raman measurements (black), with the calculated lognormal distribution (red). (**e**) Probability-probability plot visually demonstrating the goodness of fit with a lognormal distribution.

There are significant variations in the detected Raman signals across the same sample, which can be attributed to disproportionate distributions of hot spots, inconsistent functionalization of DNA strands, and fluctuations in optical setup. Here, we aim to use random sampling in surface-enhanced Raman spectroscopy to collect sufficient Raman data that provides an accurate representation of the sample population to make appropriate inferences regarding the composition within DNA strands. Thus, we use random silver films as the SERS substrates, as the substrates produce nanoscale islands that generate localized electromagnetic field enhancement while also providing a random distribution of hot-spots that limits systematic bias. We perform a Raman mapping procedure, in which we measure 400 Raman spectra across an area of approximately 100 by 100 microns of ssDNA containing 150 bases of adenine and 50 bases of cytosine (75% A/25% C) functionalized to the random silver films (Fig. 2a, methods). When qualitatively and quantitatively analyzing the data, it is apparent that there are fluctuations between each individual Raman spectrum due to the random hotspot distribution and variations in the ssDNA functionalization. As shown in Fig. 2b, five sample Raman spectra from the 75% A/25% C ssDNA show differences in the A/B and C/B ratios between each spectrum despite being acquired from the same scan. Thus, relying on a limited number of Raman spectra to extract quantitative information from the system is insufficient for the calculation of nucleic acid composition within DNA strands. Instead, we use the entire 400 Raman measurement set to accurately represent the system under study. To obtain an accurate representation of the population, we calculate the normalization ratio for each Raman spectrum in the set of 400, and then plot the probability density function (PDF) of the normalization ratio based on the 400 measurements. As an example, the probability density function of A/B ratio for the 75% A/25% C sample is shown in Fig. 2c, in which a lognormal distribution (red) is shown to be a best fit. To confirm the appropriateness of the lognormal distribution fit, we plot the cumulative density function (Fig. 2d) and the theoretical vs. empirical probabilities (Fig. 2e), which visually show the goodness of fit of the lognormal distribution for the probability distribution of the normalization ratio.

After establishing the necessary constraints to account for variations and fluctuations across samples, we propose to measure the composition within a random strand of DNA by first establishing a calibration curve using several known standards of DNA mixtures. To measure the composition of nucleic acids, ssDNA of 200 bases in length were purchased from Integrated DNA Technologies with the following compositions: (1) 200 bases of cytosine (0% A, 100% C), (2) 50 bases of adenine and 150 bases of cytosine (25% A/75% C), (3) 100 bases of adenine and 100 bases of cytosine (50% A/50% C), (4) 150 bases of adenine and 50 bases of cytosine (75% A/25% C), and (5) 200 bases of adenine (100% A, 0% C), (sequences found in Table S1).

**Figure 3:**
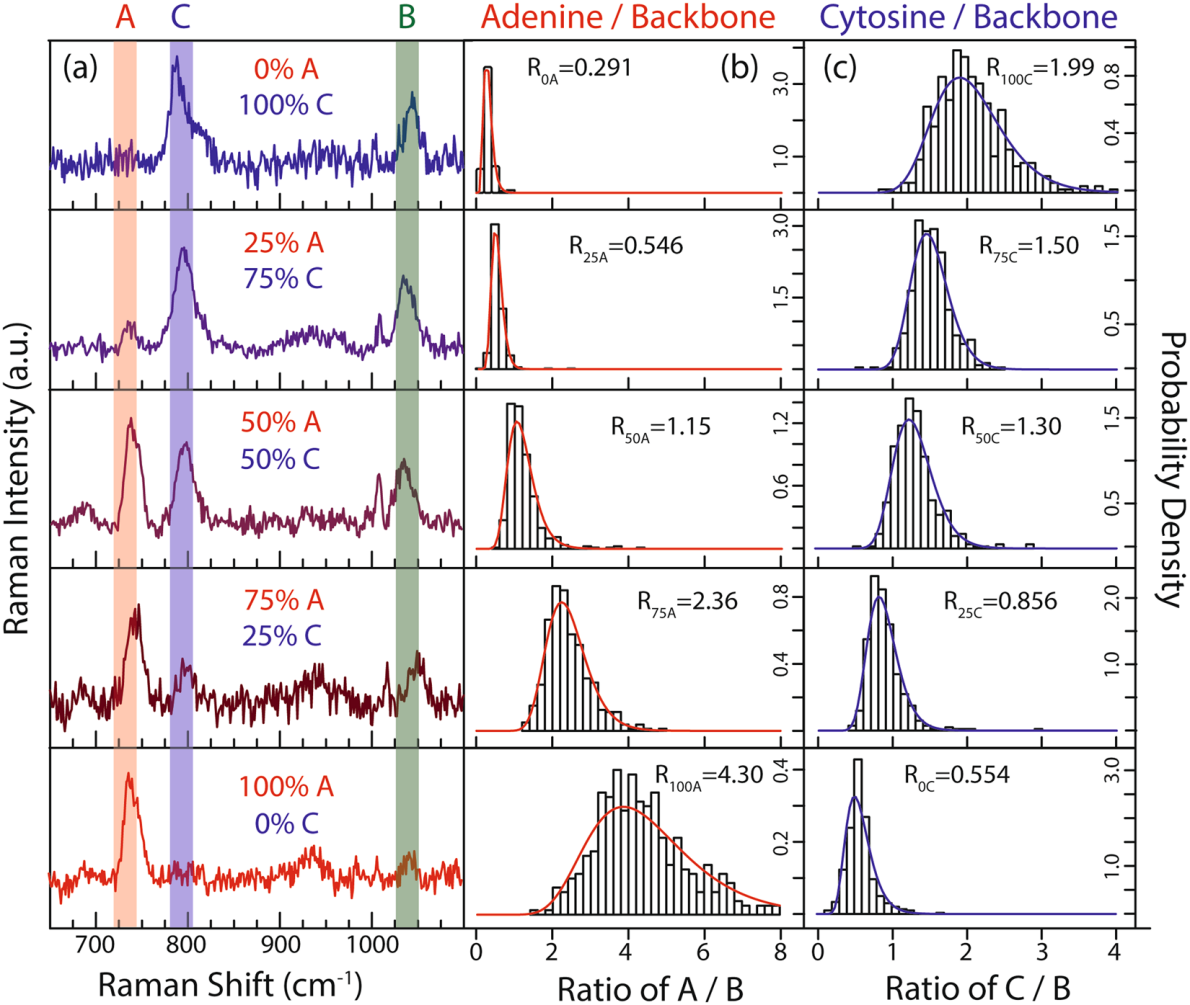
Raman spectra and corresponding probability distributions for the 5 standards. (**a**) Example Raman spectrum for each calibration standard with the adenine ring-breathing-mode (red), cytosine ring-breathing-mode (blue), and backbone mode (green) contained in the vertical shaded rectangle (see S2 for mode selection). (**b**) and (**c**) Probability density functions for the normalized ratios (A/B and C/B) for the 5 standards, showing the median of the lognormal distributions (R).

To generate the calibration curve for the estimation of composition within DNA using the random Raman sampling procedure, we perform 400 measurements on each standard and plot the probability density functions for each standard. A single Raman spectrum for each standard is shown in Fig. 3a, in which the A/B and C/B ratios are dependent on the composition of the corresponding nucleotide in the ssDNA. The PDFs for the ratios of A/B and C/B (Fig. 3b) for each standard demonstrate that the ratios for each standard follow lognormal distributions, as seen previously with the example of A/B ratio of 75% A/25% C standard in Fig. 2c–e. Incorporating 400 measurements and calculating the lognormal distribution provides more accurate information related to the composition of nucleotides in DNA strands in comparison to relying on single measurements, and thus more precise calibration curves can be generated from the Raman measurements.

**Figure 4:**
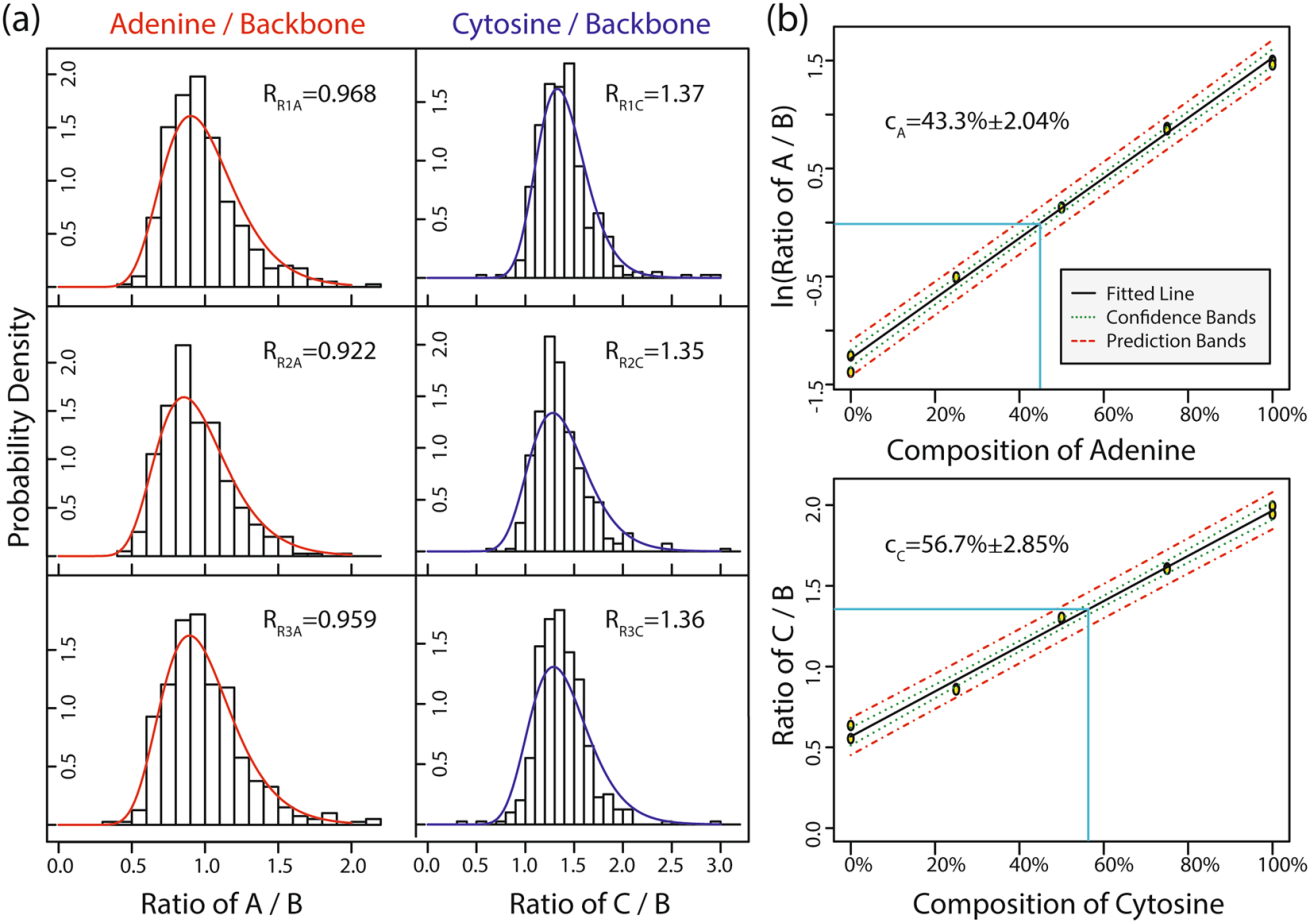
Probability density functions and calibration curve for detection of composition within random ssDNA. (**a**) PDFs of 3 sets of measurements on a random composition of ssDNA with the corresponding lognormal median of each measurement (R). (**b**) Calibration curve based on 2 sets of measurements from each standard (10 total) and the corresponding identification of the composition using the average normalization ratios of the 3 random samples.

To reduce the error in the calibration curve, two 400 Raman mapping procedures are performed for each standard with a total of 10 points (median of each lognormal distribution) used for the calibration curve. The summary of the medians of the lognormal distributions can be found in Table 1, in which it is apparent that the ratio of adenine to the backbone mode increases exponentially with respect to composition while the ratio of cytosine to the backbone mode increases linearly with respect to composition. The nonlinear dependence of the adenine normalization ratio is caused by the charge-transfer effect that generates a stronger chemical resonance^20^ with a higher composition of adenine molecules at a Raman excitation wavelength of 785 nm. The linear dependence of the cytosine normalization ratio is due to the lack of charge-transfer effect at 785 nm^21^; though it is important to note that at shorter wavelengths (e.g. 532 nm) the cytosine normalization ratio will have a nonlinear dependence. The calibration curves for both adenine and cytosine can be found in Fig. 4b, with best fit equations of ln (*R*_*A*_) = 0.0278 *C*_*A*_ − 1.25 and *R*_*C*_ = 0.0140 *C*_*C*_ + 0.567 and coefficient of determinations of *r*_*A*_ = 0.997 and *r*_*C*_ = 0.994, respectively (S4). The non-zero y-intercept is caused by the positive peak intensities between the ranges of 725 cm^−1^ and 750 cm^−1^ for adenine and 785 cm^−1^ and 810 cm^−1^ for cytosine. For adenine, the positive peak intensity is attributed to noise, while for cytosine, the positive peak intensity is caused by both noise and the existence of the phosphate skeleton stretching Raman mode of DNA which overlaps with the cytosine ring-breathing-mode. A discussion and example spectrum demonstrating the cause of the non-zero y-intercepts can be found in S2. The limits of detection (LOD) can be calculated by ${LOD}=3.3\frac{{S}_{y}}{m}$ where Sy is the standard deviation of the response and m is the slope of the calibration curve. Thus, the LOD for adenine and cytosine are compositions of 9.32% and 13.0%, respectively. To determine the composition of a random strand of DNA, we used a randomization algorithm to generate a random sequence of DNA containing 200 bases of both adenine and cytosine. The resulting ssDNA was purchased from Integrated DNA Technologies and contained 45% A and 55% C (sequence in Table S1). Three 400 Raman mapping procedures are performed on the random sample of DNA and the PDFs are calculated (Fig. 4a). Using the best fit equations, the compositions of A and C are determined to be *C*_*A*_ = 43.3% ± 2.04% and *C*_*C*_ = 56.7% ± 2.85%, respectively.

**Table 1:** Median of the lognormal distributions for each standard and the random sample.

Measurement	Lognormal Median of A/B	Lognormal Median of C/B
0% A/100% C #1	0.291	1.99
0% A/100% C #2	0.250	1.94
25% A/75% C #1	0.546	1.50
25% A/75% C #2	0.598	1.61
50% A/50% C #1	1.15	1.30
50% A/50% C #2	1.14	1.31
75% A/25% C #1	2.36	0.856
75% A/25% C #2	2.41	0.864
100% A/0% C #1	4.30	0.554
100% A/0% C #2	4.49	0.635
Random Sample #1	0.968	1.37
Random Sample #2	0.922	1.35
Random Sample #3	0.959	1.36

## Discussion

**Figure 5:**
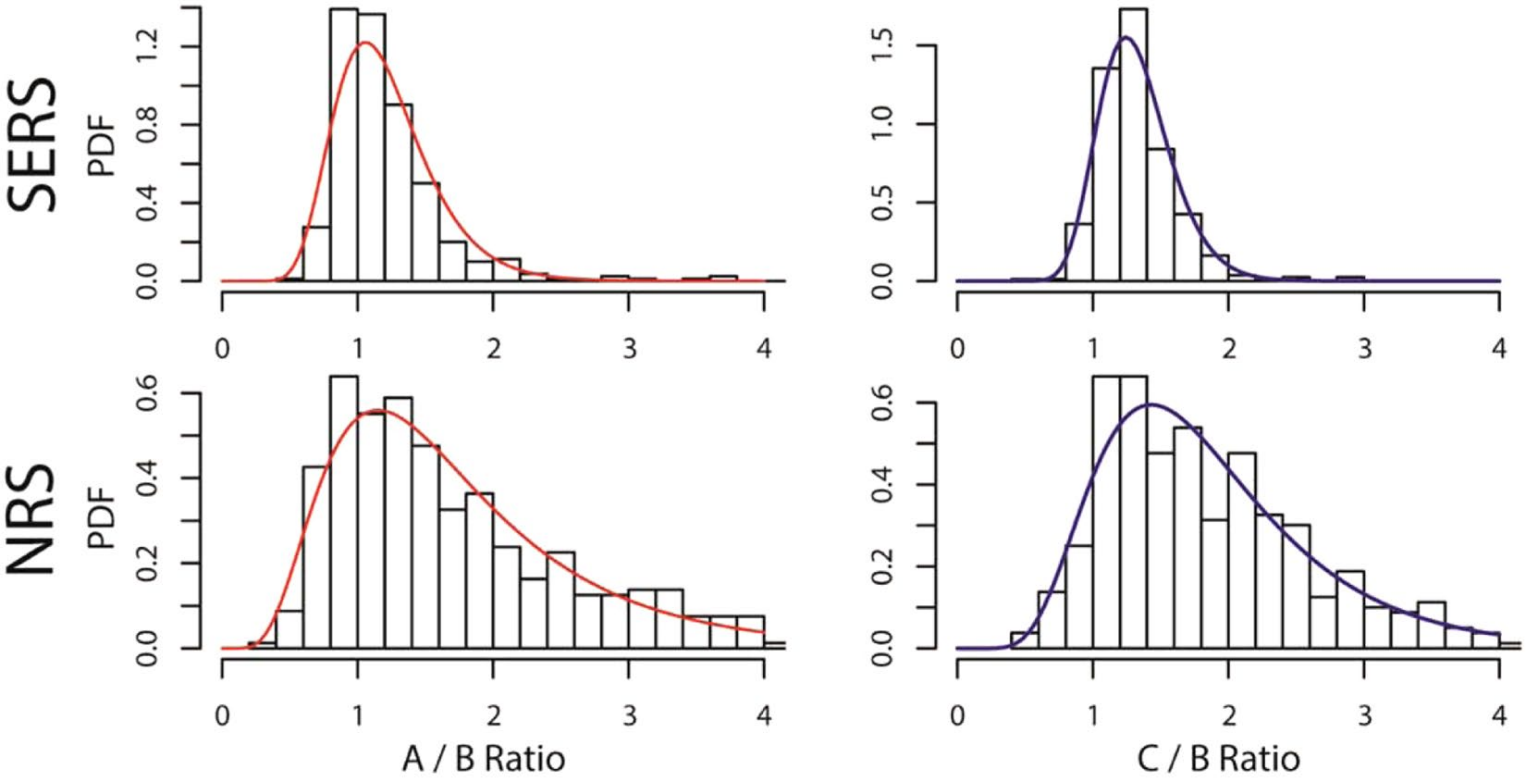
Comparison of SERS and NRS for the 50% A/50% C standard, showing a tighter distribution for the SERS sample.

Here, we have shown the feasibility of calculating the composition of DNA strands with a standard error of ± 2.04% and ± 2.85% for A and C, respectively, using cost efficient random silver films and a low concentration of DNA. To qualitatively compare this technique to a random Raman mapping procedure without SERS, we functionalized the ssDNA to mica and acquired the normal Raman scattering (NRS) spectra which results in a poor signal to noise ratio and greater variance in the A/B and C/B ratios. Using the 50% A/50% C standard, the average peak intensities of the A and C ring-breathing-modes for the normal Raman scattering spectra set are measured at 71.9 a.u. and 73.4 a.u., respectively. This is lower than the average peak intensities of the A and C ring-breathing-modes for the SERS spectra set, which are 468 a.u. and 510 a.u., respectively. Thus, the surface-enhanced affect improves the signal to noise ratio and also reduces the variation in the measured signals, allowing for a lower standard error in the calibration curves. To visualize this difference, Fig. 5 shows the population distributions with the fitted lognormal probability density functions for the A/B and C/B ratios of the normal Raman scattering (NRS) spectra set and the surface-enhanced Raman scattering (SERS) spectra set. The larger variances of NRS (1.05 for A/B and 0.847 for C/B) compared to the variances of SERS (0.428 for A/B and 0.340 for C/B) suggest that the standard error for NRS will be much greater than that of SERS. This also suggests that incorporating a SERS substrate with a greater enhancement will reduce the variance and minimize the standard error.

Thus, we have demonstrated that our method is a great improvement over performing these measurements using normal Raman scattering without enhancement from a SERS substrate. To further improve our method to achieve single nucleotide resolution in the future, we propose to implement high local field enhancement factor nanoplasmonic resonators^22–24^ that will greatly enhance the signal to noise ratio of the system. Despite the cost efficiency of random silver films, they are poor SERS substrates due to their weak local field enhancement with LFE estimations between 0.9 to 2.5, depending on fill factor and excitation wavelength^25^. We can aim for the utilization of resonators capable of highly confining the local electric field, which will increase the LFE from a maximum of 2.5 to a range of 10–100^26,27^. Using plasmonic resonators capable of LFE between 10 to 100 would drastically increase the signal to noise ratio, which in turn would reduce our standard error to a level capable of achieving single nucleotide resolution.

In this work, we demonstrated label-free detection of the adenine and cytosine composition within DNA strands using a random Raman mapping procedure. Single strands of DNA composed of adenine and cytosine were functionalized to random silver films, and 400 Raman measurements were acquired for 5 different standards. The normalization ratio of the adenine and cytosine to backbone modes were calculated for each set of measurements, and the probability density functions were calculated which fit lognormal distributions. Calibration curves were generated using the standards, and the nucleotide composition of a random sequence of ssDNA was estimated. Thus, this work shows promise in implementing this novel method as a technique in determining the composition of bases within DNA strands without the use of labels.

While the work discussed here is promising, additional method development is necessary to realize eventual application for DNA composition detection. This work focused on adenine and cytosine as a proof of concept, so future direction will consist of determining the composition of all bases within random strands of DNA. This may present some technical challenges as the thymine ring-breathing-mode at ~800 cm^−1^ overlaps with the cytosine ring-breathing-mode at 795 cm^−1^, so the signals must be deconvoluted from each other. Additional future developments will incorporate higher local field enhancement nanoplasmonic resonators that will increase the signal to noise ratio, reduce variation in the Raman spectra, and minimize standard error in the calibration curve. Eventually, the aim of the technique is to utilize this method for future label-free studies of DNA, in which distinctions can be made on changes to the DNA composition without the need to modify the DNA using standard techniques such as labeling assays.

## Methods

For the experimental measurements, silver films are fabricated by depositing 300 Å of silver using an e-beam evaporator (Temescal BJD, UCSD Nano3 Cleanroom) on a mica substrate. To functionalize the ssDNA to the random silver films, ssDNA solutions were prepared by diluting the DNA stock solution to 50 ng/uL in HEPES and then forming a 1:1 mixture of the ssDNA solution to 10 mM magnesium chloride. A table of the sequences for each ssDNA standard can be found in the supplementary information. The DNA concentration selected was chosen to ensure sufficient coverage, and the corresponding salt ratio was used to neutralize the phosphate backbone and promote attachment of the nucleotides directly to the silver. 20 uL of each solution was deposited onto separate substrates of random silver films for 25 minutes, followed by rinsing with buffer and drying under nitrogen gas to remove multilayers of DNA.

Raman spectra were acquired using a Renishaw inVia Raman spectrometer, using a 785 nm 500 mW continuous wave laser at a power of 1% and an acquisition time of 3 s. The grating was set to static with a spectrum range of ~100 cm^−1^ to 1100 cm^−1^ for each measurement. The Raman mapping feature was used to acquire 400 measurements in a 20 × 20 unit area, with each unit representing an area of ~ 25 µm^2^. After acquiring the 400 measurements, cosmic ray removal and baseline subtraction using the Intelligent Fitting function were performed using the Renishaw WiRE 4.2 software. MATLAB was used to find the peak intensity for the ring-breathing-modes of adenine (between 725 cm^−1^ and 750 cm^−1^) and cytosine (between 785 cm^−1^ and 810 cm^−1^) and the phosphate backbone mode of DNA (between 1020 cm^−1^ and 1045 cm^−1^) for each of the 400 spectra. The A/B and C/B histograms of the 400 measurements was plotted in R, and the lognormal distribution fit parameters were calculated using the fitdistrplus package. Additional information on the fitting models can be found in the supplementary information.

## Electronic supplementary material

Supplementary Information
