# Supplementary material for: Self-reference and random sampling approach for label-free identification of DNA composition using plasmonic nanomaterials
Source: Sci Rep. 2018 May 9;8:7398. doi: 10.1038/s41598-018-25444-2 (PMC5943445; doi:10.1038/s41598-018-25444-2)
Supplement: Supplementary file 1 — Supplementary Information [file 41598_2018_25444_MOESM1_ESM.docx]

**Self-reference and random sampling approach for label-free identification of DNA composition using plasmonic nanomaterials**

Lindsay M. Freeman, Lin Pang, Yeshaiahu Fainman

**Content of Sections**

**S1. Sequences of DNA**

**S2. Signal Processing of Raman Measurements**

**S3. Probability Density Functions with Lognormal Distribution Fits**

**S4. Statistical Calculations**

**S1. Sequences of DNA**

Table S1.1. Sequences of ssDNA used in experiments

| Oligonucleotide | Sequence (5’—3’) |
| --- | --- |
| 0% A / 100% C Standard | CCC CCC CCC CCC CCC CCC CCC CCC CCC CCC CCC CCC CCC CCC CCC CCC CCC CCC CCC CCC CCC CCC CCC CCC CCC CCC CCC CCC CCC CCC CCC CCC CCC CCC CCC CCC CCC CCC CCC CCC CCC CCC CCC CCC CCC CCC CCC CCC CCC CCC CCC CCC CCC CCC CCC CCC CCC CCC CCC CCC CCC CCC CCC CCC CCC CCC CCC CCC CCC CCC CCC CC |
| 25% A / 75% C Standard | AAA AAC CCC CCC CCC CCC CCA AAA ACC CCC CCC CCC CCC CAA AAA CCC CCC CCC CCC CCC AAA AAC CCC CCC CCC CCC CCA AAA ACC CCC CCC CCC CCC CAA AAA CCC CCC CCC CCC CCC AAA AAC CCC CCC CCC CCC CCA AAA ACC CCC CCC CCC CCC CAA AAA CCC CCC CCC CCC CCC AAA AAC CCC CCC CCC CCC CC |
| 50% A / 50% C Standard | AAA AAA AAA ACC CCC CCC CCA AAA AAA AAA CCC CCC CCC CAA AAA AAA AAC CCC CCC CCC AAA AAA AAA ACC CCC CCC CCA AAA AAA AAA CCC CCC CCC CAA AAA AAA AAC CCC CCC CCC AAA AAA AAA ACC CCC CCC CCA AAA AAA AAA CCC CCC CCC CAA AAA AAA AAC CCC CCC CCC AAA AAA AAA ACC CCC CCC CC |
| 75% A / 25% C Standard | CCC CCA AAA AAA AAA AAA AAC CCC CAA AAA AAA AAA AAA ACC CCC AAA AAA AAA AAA AAA CCC CCA AAA AAA AAA AAA AAC CCC CAA AAA AAA AAA AAA ACC CCC AAA AAA AAA AAA AAA CCC CCA AAA AAA AAA AAA AAC CCC CAA AAA AAA AAA AAA ACC CCC AAA AAA AAA AAA AAA CCC CCA AAA AAA AAA AAA AA |
| 100% A / 0% C Standard | AAA AAA AAA AAA AAA AAA AAA AAA AAA AAA AAA AAA AAA AAA AAA AAA AAA AAA AAA AAA AAA AAA AAA AAA AAA AAA AAA AAA AAA AAA AAA AAA AAA AAA AAA AAA AAA AAA AAA AAA AAA AAA AAA AAA AAA AAA AAA AAA AAA AAA AAA AAA AAA AAA AAA AAA AAA AAA AAA AAA AAA AAA AAA AAA AAA AAA AA |
| Random Mixture | AAC AAA CCC ACC ACA CCA ACA CAA CCC CAC CCC ACC CAA CAA CCA AAC CCC AAA ACC CCC CAC AAC CAA CCA AAA AAC AAC CAA CAC CCA ACC ACA CCC ACA CAA CAC CCA AAA CAC CAC CCC AAC AAC CCC ACC CCA CCC ACA CAA CAC CAA ACA CCC CAA CCA ACA CAA CCC ACA CCC ACC CAA CAA CCC CAA CAC AC |

**S2. Signal Processing of Raman Measurements**

Because no smoothing filter is used when analyzing the spectra, there is noise present in the spectra that results in non-zero A / B and C / B ratios in the adenine mode of 0% A / 100% C and in the cytosine mode of 100% A / 0% C, respectively. Figure S2.1 shows the noise for the adenine and backbone modes of the 100% A / 0% C, 50% A / 50% C, and 0% A / 100% C mixtures, with the peak selection range displayed by the vertical gray dotted lines. The peak intensity between each range is shown in the upper left corner of the adenine mode and upper right corner of the backbone mode. Using these values, we determine A / B ratios of 2.91 (983 a.u. / 338 a.u.), 1.02 (305 a.u. / 299 a.u.), and 0.290 (92.9 a.u. / 321 a.u.) for the 100% A / 0% C, 50% A / 50% C, and 0% A / 100% C mixtures, respectively, demonstrating the cause of non-zero intercepts in mixtures containing 0% of adenine or cytosine.

Figure S2.1. Segments of Raman spectra emphasizing the peak intensities between each range


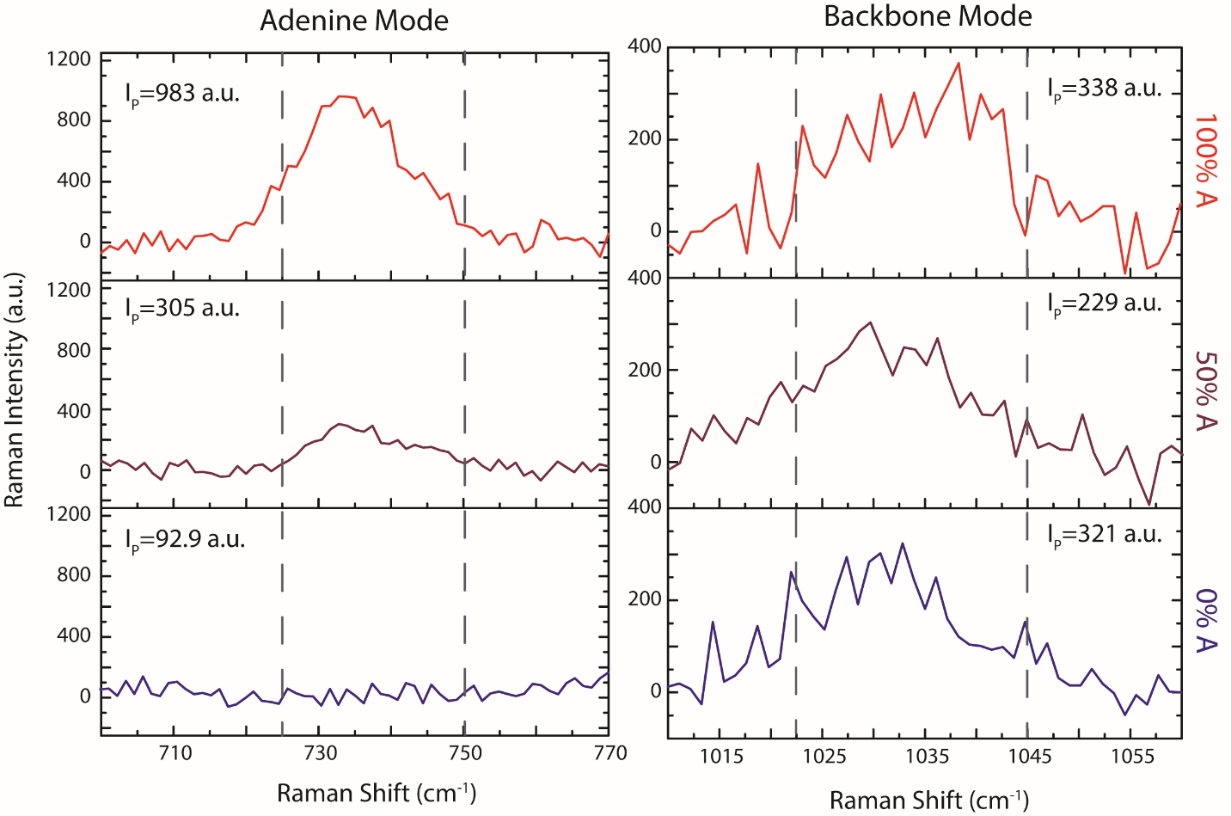


**S3. Probability Density Functions with Lognormal Distribution Fits**

The probability distribution histograms for each set of 400 measurements of the ratio of A / B or C / B were plotted in R. Using the fitdistrplus package, various probability density functions (PDFs) were fit to the histogram data and visually compared to the histogram. The break feature was used to select the binning depending on the distribution of the data, ranging from small break sizes of 5 for tight distributions (e.g. A / B ratio of 0% adenine standard) to larger break sizes of 75 for broad distributions (e.g. A / B ratio of 100% adenine standard). To determine the PDFs, as an example in Figure S3.1, four probability density functions (normal, lognormal, gamma, and weibull) calculated from the histogram data of the A / B ratio from the 25% A / 75% C standard are plotted. The corresponding cumulative distribution functions (CDFs) and probability-probability (P-P) plots are shown for each fit. The PDF defines the continuous probability distribution, the CDF is the discrete distribution of the data points, and the P-P plot shows the comparison of the empirical CDF to the theoretical PDF (e.g. normal, lognormal) which aids in visualizing whether the PDF is a good fit to the CDF. A visual comparison of the PDF, CDF, and P-P plots demonstrate that the lognormal density function is the best fit^1^.

From this data, we can see that an increase in the percent composition of a nucleotide leads to a broadening of the full width at half maximum due to the larger variance of the nucleotide Raman intensity mode. This leads to a decrease in the density of probability, as a broader distribution leads to lower probability at each bin.

.

Figure S3.1. PDFs, CDFs, and P-P plots for normal, lognormal, gamma, and weibull fits of the A / B ratio of the 25% A / 75% C mixture


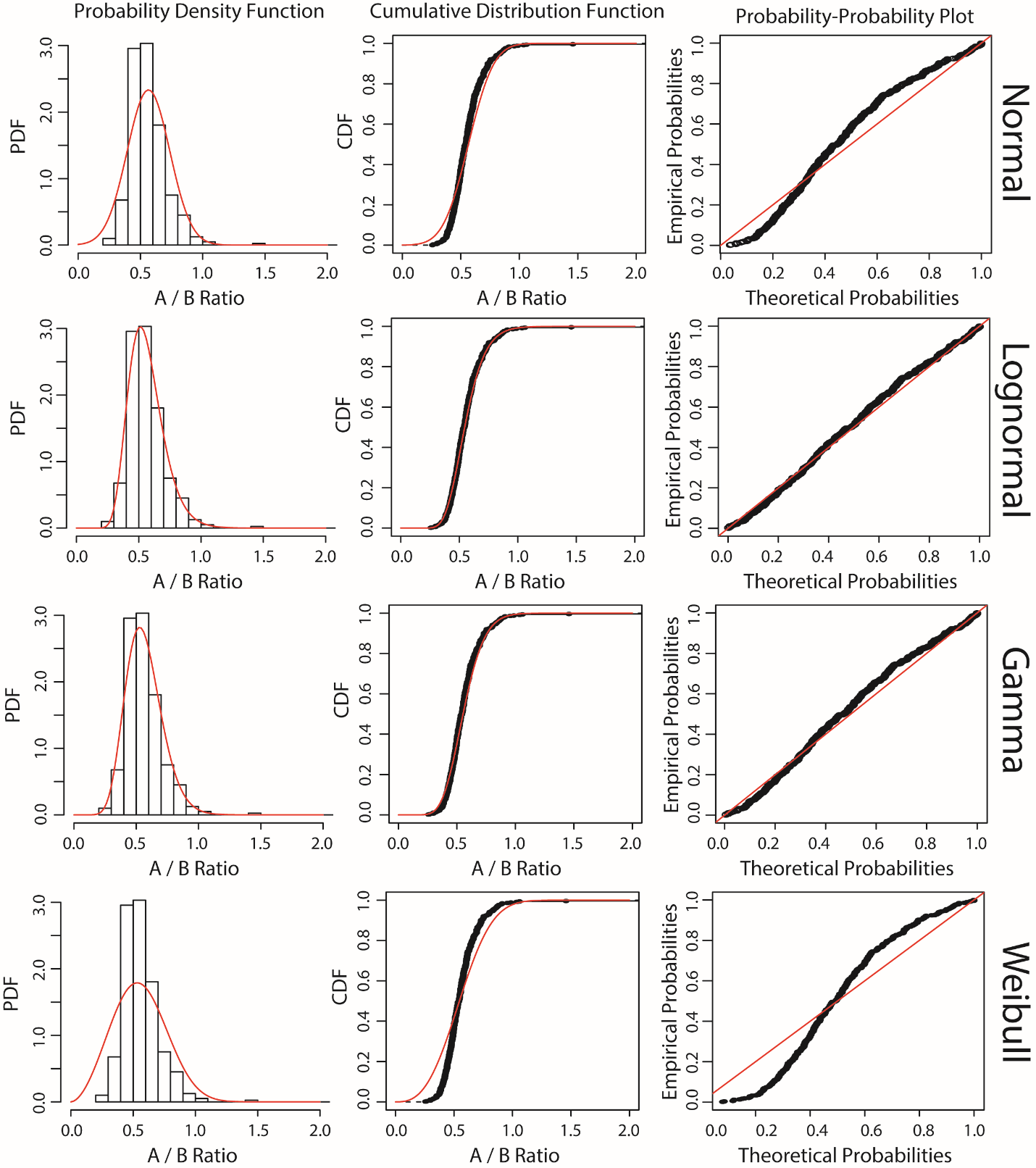


Upon determining that the lognormal distribution is the best fit for these datasets, the PDFs, CDFs, and P-P plots for the lognormal distribution are calculated for each measurement. As explained in the main text, 5 standards were used, in which 2 Raman maps were taken of each standard. This results in a total of 10 sets of 400 measurements. The complete results are found in Figures S3.2-S3.6.

Figure S3.2. Probability density function, cumulative distribution function, and probability-probability plots of the A / B and C / B ratios of the 0% A / 100% C mixture


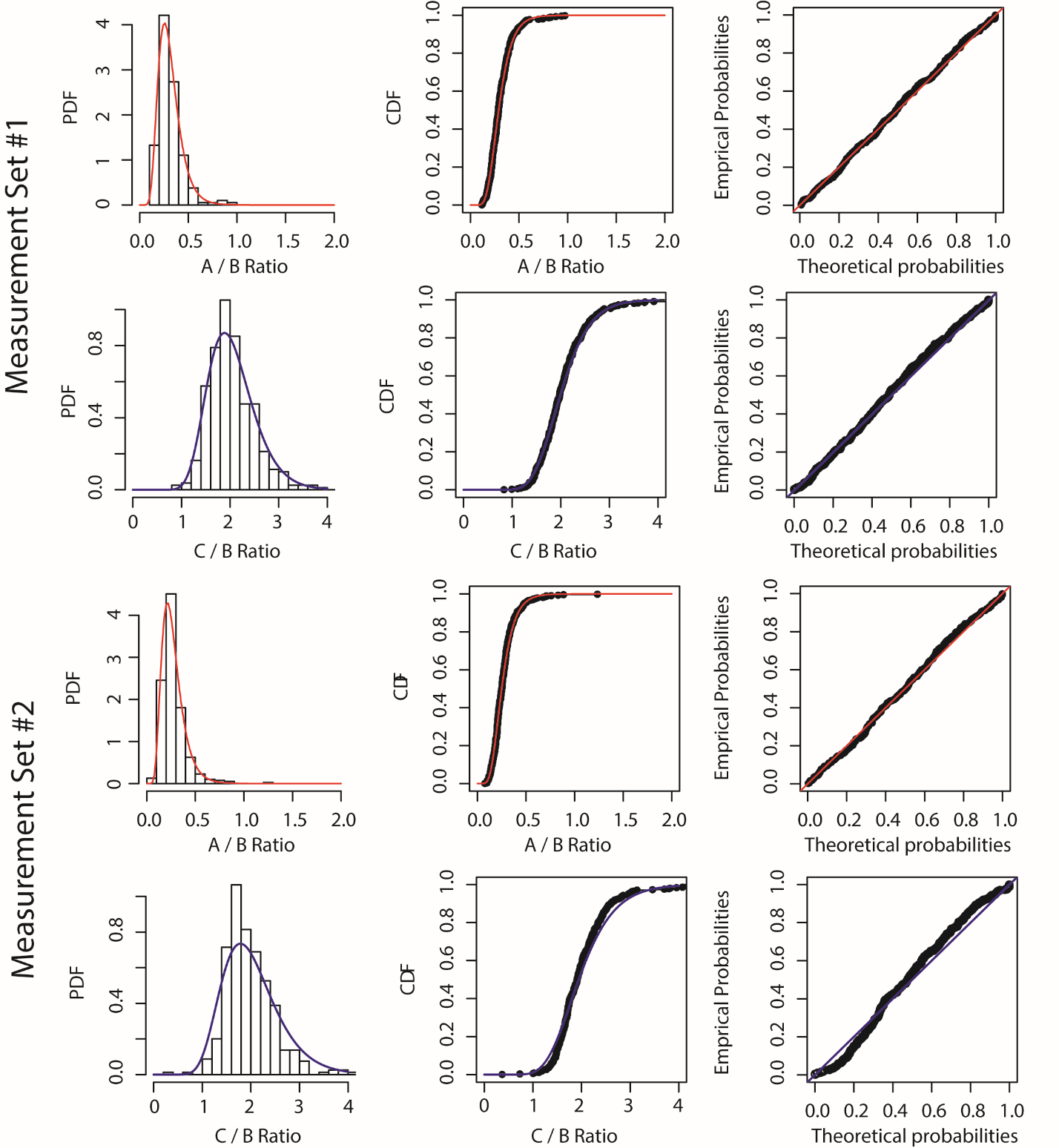


Figure S3.3. Probability density function, cumulative distribution function, and probability-probability plots of the A / B and C / B ratios of the 25% A / 75% C mixture


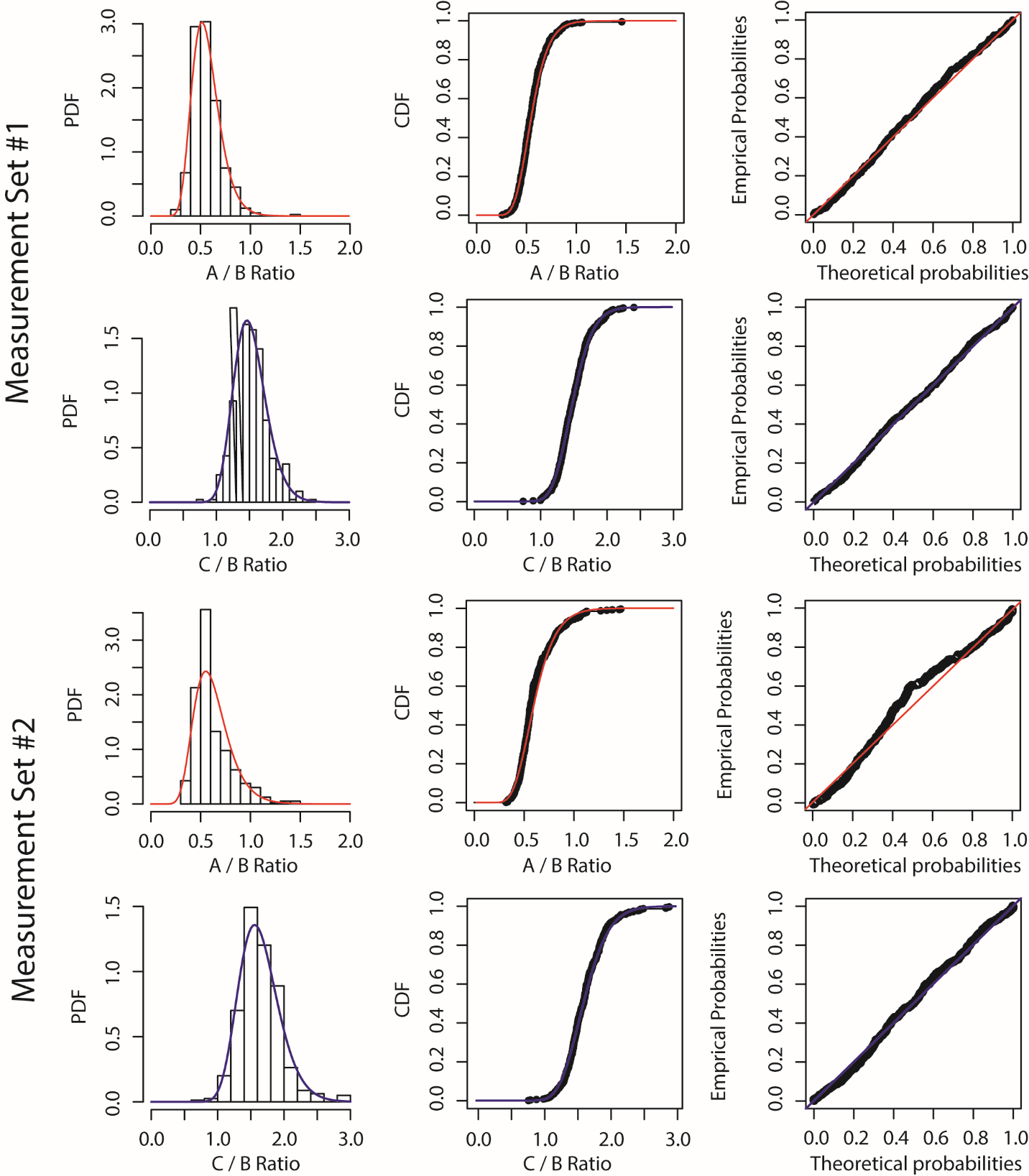


Figure S3.4. Probability density function, cumulative distribution function, and probability-probability plots of the A / B and C / B ratios of the 50% A / 50% C mixture


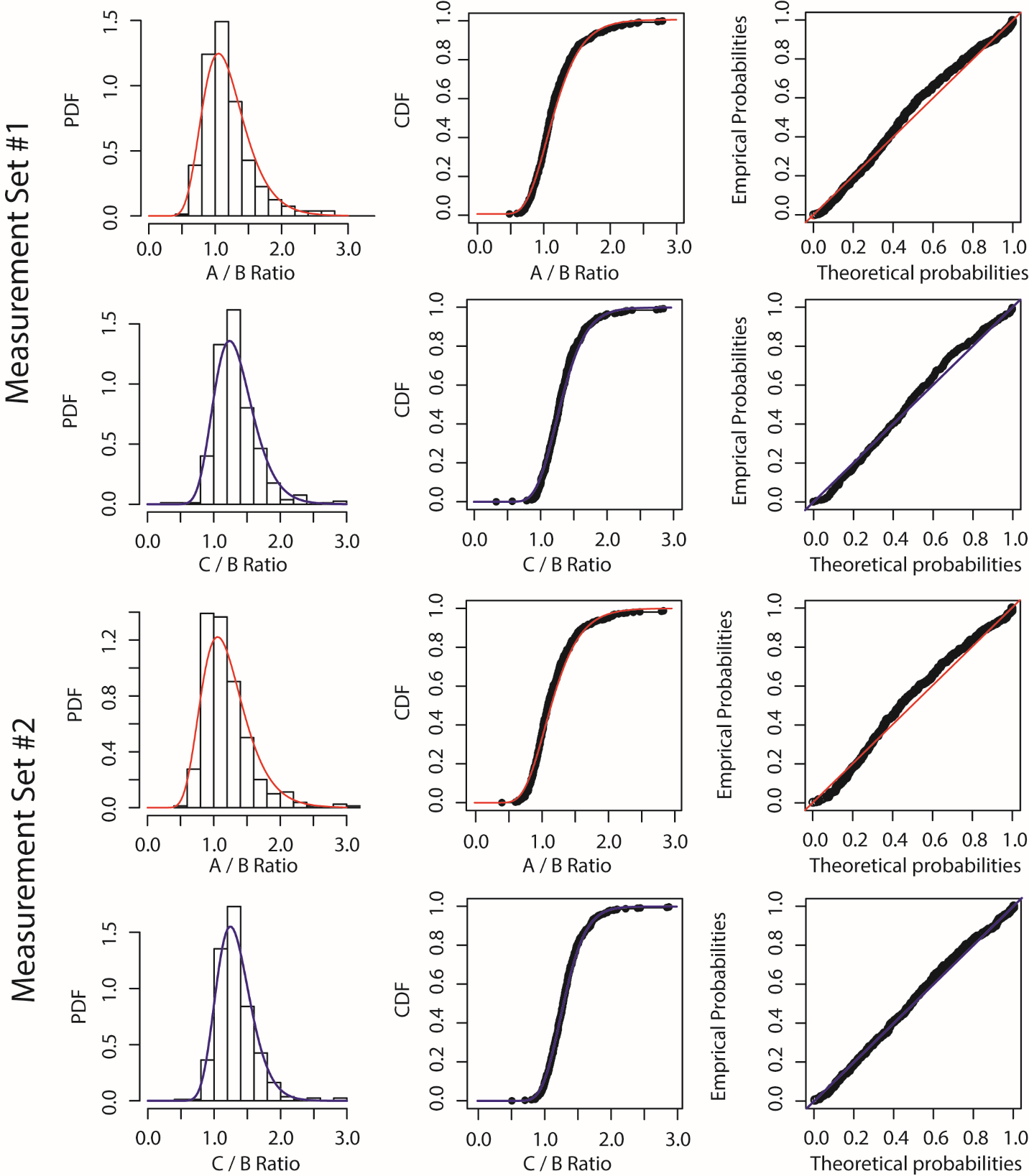


Figure S3.5. Probability density function, cumulative distribution function, and probability-probability plots of the A / B and C / B ratios of the 75% A / 25% C mixture


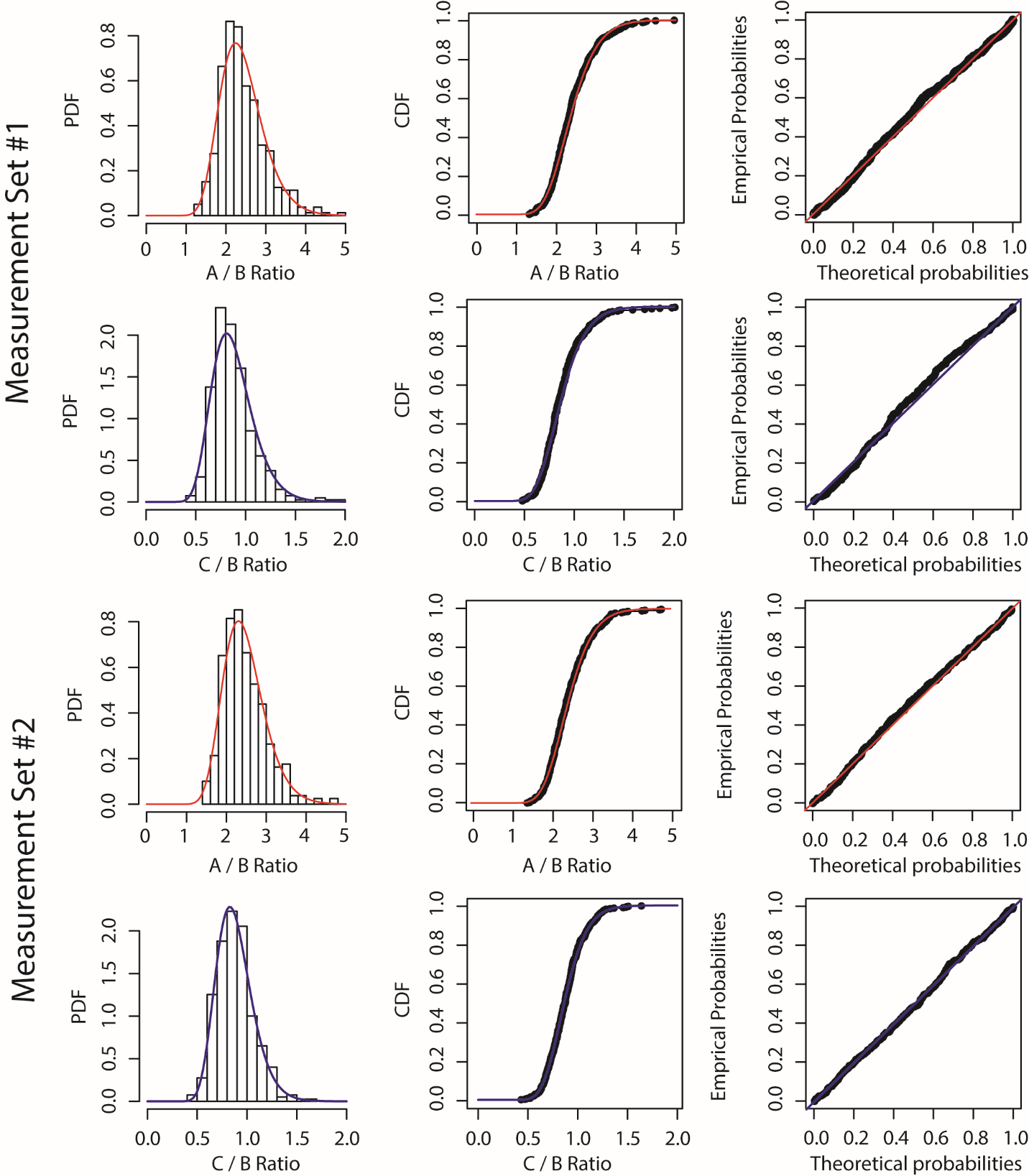


Figure S3.6. Probability density function, cumulative distribution function, and probability-probability plots of the A / B and C / B ratios of the 100% A / 0% C mixture


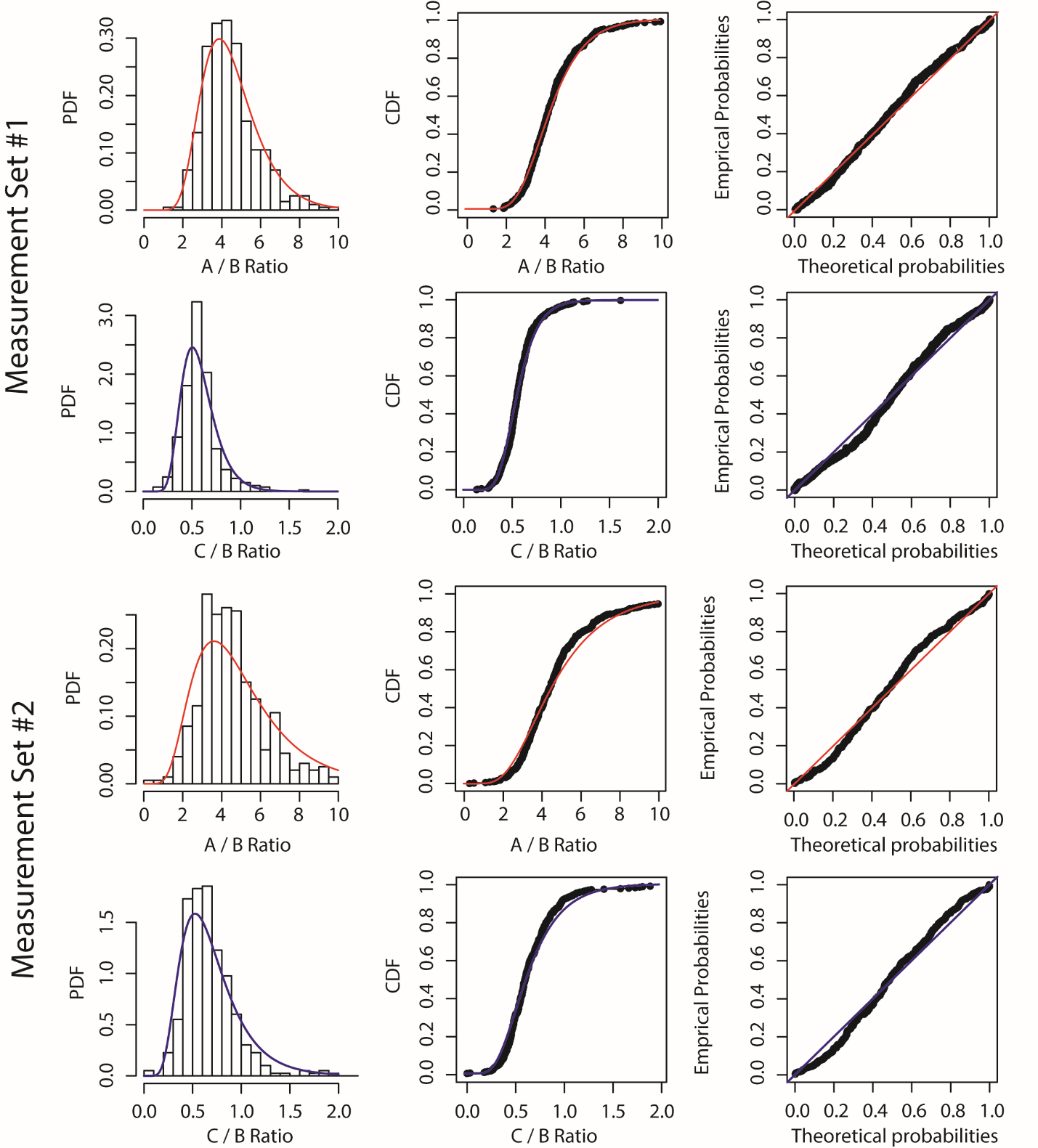


**S4. Statistical Calculations**

The compositions and corresponding lognormal median of the ratios (A / B for adenine and C / B for cytosine) can be found in table S4.1.

Table S4.1. Compositions and lognormal median of the ratios for adenine and cytosine

| **C_A_** | **R_A_** | **ln(R_A_)** | **C_C_** | **R_C_** |
| --- | --- | --- | --- | --- |
| 0 | 0.250 | -1.39 | 100 | 1.99 |
| 0 | 0.291 | -1.24 | 100 | 1.94 |
| 25 | 0.546 | -0.514 | 75 | 1.61 |
| 25 | 0.598 | -0.504 | 75 | 1.6 |
| 50 | 1.14 | 0.134 | 50 | 1.3 |
| 50 | 1.15 | 0.143 | 50 | 1.31 |
| 75 | 2.36 | 0.859 | 25 | 0.864 |
| 75 | 2.41 | 0.879 | 25 | 0.856 |
| 100 | 4.30 | 1.46 | 0 | 0.635 |
| 100 | 4.49 | 1.50 | 0 | 0.554 |

To calculate the best fit equation with error for the calibration curve, the mean composition ($\bar{C}$) and mean signal ($\bar{R}$) are calculated according to the following equations:

$\bar{C}=\frac{\sum_{i=1}^{n} C_{i}}{n}$ S4.1

$\bar{R}=\frac{\sum_{i=1}^{n} R_{i}}{n}$ S4.2

Then, to ensure the best fit equation has strong correlation (r>0.99), the r value is calculated according to the following equation:

$r=\frac{\sum_{i} [\left( C_{i}-\bar{C} \right)\left( R_{i}-\bar{R} \right)]}{\sqrt{\sum_{i} \left( C_{i}-\bar{C} \right)^{2}\sum_{i} \left( R_{i}-\bar{R} \right)^{2}}}$ S4.3

in which r_A_ = 0.997 and r_C_ = 0.994. Then the slope (m) and y-intercept (b) of the calibration curve can be found by:

$m=\frac{\sum_{i} [\left( C_{i}-\bar{C} \right)\left( R_{i}-\bar{R} \right)]}{\sum_{i} \left( C_{i}-\bar{C} \right)^{2}}$ S4.4

$b=\bar{R}-m\bar{C}$ S4.5

$R=b+mC$ S4.6

resulting in best fit equations of the calibration curves of $R_{A}=0.0278C_{A}-1.25$ and $R_{C}=0.0140C_{C}+0.567$ for adenine and cytosine, respectively.

To calculate the error, the residuals are found via the following equation:

$\hat{R}_{i}=b+mC_{i}$ S4.7

The standard deviation of the signal based on the residuals is found by:

$\sigma_{R/C}=\sqrt{\frac{\sum_{i} \left( R_{i}-\hat{R}_{i} \right)^{2}}{n-2}}$ S4.8

The confidence limits for the slope and y-intercept can then be calculated by first finding the standard deviation:

$\sigma_{m}=\frac{\sigma_{R/C}}{\sqrt{\sum_{i} \left( C_{i}-\bar{C} \right)^{2}}}$ S4.9

$\sigma_{b}=\sigma_{R/C}\sqrt{\frac{\sum_{i} C_{i}^{2}}{n\sum_{i} \left( C_{i}-\bar{C} \right)^{2}}}$ S4.10

At a 90% confidence interval (alpha = 0.1) and 9 degrees of freedom (DOF = n-1), the t-value is 1.383. The slope and y-intercept equations with confidence limits can then be calculated with the following formulas:

$m_{CL}=m\pm t\sigma_{m}$ S4.11

$b_{CL}=b\pm t\sigma_{b}$ S4.12

This results in the slopes and y-intercepts with confidence limits of $m_{A}=0.0278\pm0.000761$ and $b_{A}=-1.25\pm0.0466$ for adenine and $m_{C}=0.0140\pm0.000535$ and $b_{C}=0.567\pm0.0328$ for cytosine.

When measuring the signal of an unknown composition and using the calibration curve to calculate the composition, the following equations are used:

$c_{sample}=\frac{b+R}{m}$ S4.13

$\sigma_{sample}=\frac{\sigma_{R/C}}{m}=\sqrt{\frac{1}{k}+\frac{1}{n}+\frac{\left( R_{sample}-\bar{R} \right)^{2}}{m^{2}\sum_{i} \left( C_{i}-\bar{C} \right)^{2}}}$ S4.14

Where k is the number of measurements on the random sample (k=3). The final composition of the unknown sample can be calculated from:

$C_{sample}=\frac{b+R}{m}\pm t\sigma_{sample}$ S4.15

With a t-value of 1.383, the estimated compositions are $C_{A}=43.3\%\pm2.04\%$ and $C_{C}=56.7\%\pm2.85\%$ for adenine and cytosine, respectively.
